# Decreased Autophagy Impairs Decidualization of Human Endometrial Stromal Cells: A Role for ATG Proteins in Endometrial Physiology

**DOI:** 10.3390/ijms20123066

**Published:** 2019-06-23

**Authors:** Ana Cecilia Mestre Citrinovitz, Thomas Strowitzki, Ariane Germeyer

**Affiliations:** Department of Gynecological Endocrinology and Fertility Disorders, Women’s Hospital, Ruprecht-Karls University of Heidelberg, INF 440, 69120 Heidelberg, Germany; Thomas.Strowitzki@med.uni-heidelberg.de (T.S.); Ariane.Germeyer@med.uni-heidelberg.de (A.G.)

**Keywords:** endometrial stromal cells, decidualization, autophagy, ATG7, ATG5

## Abstract

During the menstrual cycle, the endometrium undergoes cyclic changes of cellular proliferation, differentiation, and death, an essential preparation of the endometrium for its interaction with the implanting embryo. In particular, the differentiation of endometrial stromal cells, named decidualization, ensures the formation of a proper feto-maternal interface for a regulated trophoblast invasion and correct placental orientation and growth. Interestingly, autophagy, an intracellular degradation process of great importance for the maintenance of cellular homeostasis, plays an important role in cell proliferation, differentiation, and growth. In the endometrium, increased detection of autophagy markers correlates with the progression of the menstrual cycle. However, until now, it was unknown whether autophagy contributes to the proper function of the endometrium. In this study, we show that autophagy is increased during in vitro decidualization of human endometrial stromal cells. Furthermore, we demonstrate that the knockdowns of two important autophagy-related (ATG) proteins, ATG7 and ATG5, impaired decidualization, confirming a positive role of these proteins and of autophagy for the correct decidualization of human endometrial stromal cells. In conclusion, in this work, we describe a previously unknown functional connection between autophagy and endometrial physiology.

## 1. Introduction

The inner lining of the uterus, known as the endometrium, undergoes cyclic rounds of cellular proliferation, differentiation, and death defining each stage of the menstrual cycle. These cyclic changes in cellular fate are tightly regulated by plasma levels of the ovarian steroid hormones, progesterone (P) and estradiol (E), and by the activation and crosstalk with the signaling pathway of cyclic adenosine monophosphate (cAMP) [1,2,3]. Endometrial changes are necessary to prepare the endometrium for its interaction with the implanting embryo. Among these changes, the differentiation of endometrial stromal cells (ESC) is of great importance to ensure the formation of a proper feto-maternal interface and to guide trophoblast invasion and placental orientation and growth [3,4].

The differentiation of ESC, named decidualization, comprises a complex change in cell morphology and function. During decidualization, fibroblast-like ESC expand their cytoplasm and nucleus becoming large specialized secretory cells. Decidualized cells are characterized by the accumulation of glycogen and lipids droplets in their cytoplasm and by the presence of numerous lysosomes [5,6]. Furthermore, the endoplasmic reticulum, a hallmark of secretory cells, becomes highly developed to synchronize with the intense secretory role of decidual cells. Two of the main secretory products of decidual cells are prolactin (PRL) and Insulin-like growth factor binding protein 1 (IGFBP1). Both proteins are commonly used as decidualization markers [2,7].

Macroautophagy (herein referred to as autophagy, meaning “self-eating”) is a highly conserved intracellular degradation pathway that contributes to the maintenance of cellular homeostasis. Interestingly, it has been described that autophagy plays an important role during embryo development and cellular proliferation, differentiation, and growth [8]. During autophagy, a double-membrane vesicle, the autophagosome, engulfs cytoplasmic material to deliver it to the lysosomes for degradation and recycle. The engulfment of cytoplasmatic components can be selective (selective autophagy) or non-selective (bulk autophagy) [9]. The proteins involved in autophagosome formation and in the autophagy pathway are known as autophagy-related (ATG) proteins, and the proteins related to cargo recognition are referred to as autophagy receptors [10,11]. Dysregulation of autophagy and related pathways has been associated with several diseases, including different types of cancer and neurodegenerative conditions [12,13,14].

There is increasing evidence that connects autophagy with endometrial health [15]. In the endometrium, the increased detection of the lipidated form of the microtubule-associated protein light chain 3 B (LC3BII), an autophagosome marker, correlates with the progression of the menstrual cycle, reaching its maximum towards the late secretory phase [16]. In addition, previous studies suggest that a decreased decidualization in obese mice could be related to impaired autophagy [17]. Furthermore, differences in the expression of autophagy markers between normal and abnormal villous and decidual samples from early pregnancies have been described [18]. Finally, autophagy has been associated with the onset of endometriosis, a benign fertility-related pathology [19].

Besides the evidence cited above, until now, it was unknown whether autophagy contributes to the proper function of the endometrium. The aim of this study was to evaluate if autophagy is required for the correct decidualization of human ESC. In this study, we show that autophagy is increased during the decidualization of an immortalized cell line of human ESC (t-HESC). Furthermore, we demonstrate that the knockdowns of two important ATG proteins, ATG7 and ATG5, impair decidualization confirming a positive role of these ATG proteins and of autophagy for the correct decidualization of human endometrial stromal cells.

## 2. Results

### 2.1. Autophagic Flux is Increased During t-HESC Decidualization

The first goal of this study was to evaluate whether autophagic flux (AF) is modified during the decidualization of t-HESC, a cell line that has a normal response to decidual stimulation [20]. We focused on analyzing AF during early times of the decidualization treatment as stressful culture conditions, such as overcrowding, could also activate autophagy leading to misinterpretation of the obtained results [21].

**Figure 1:**
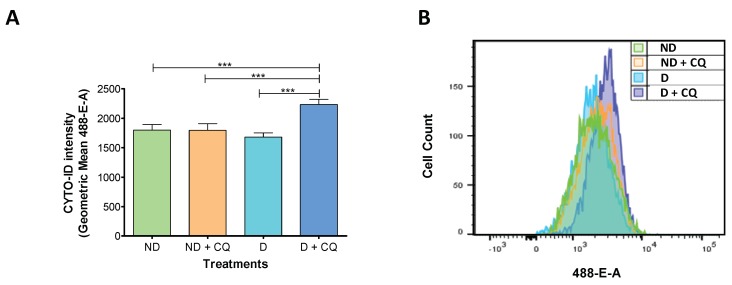
Changes in autophagic flux during decidualization. Immortalized human endometrial stromal cells (t-HESC) were treated with or without MPA + E + 8-Br-cAMP for 48 h. Chloroquine (25 µM) was added 6 h before harvesting the cells. (**A**) Flow cytometry analysis of t-HESC stained with CYTO-ID Autophagy detection kit. Data represent the geometric mean of CYTO-ID intensity ± SEM (*n* = 8). (**B**) Representative cell count profile from experiments quantified in A. *** *p* < 0.001 by one-way ANOVA followed by Tukey test. ND: non-decidualized, D: decidualized; CQ: chloroquine; t-HESC: immortalized human endometrial stromal cells; MPA: medroxyprogesterone; E: estradiol; 8-Br-cAMP: 8-Bromo-cyclic adenosine monophosphate.

AF was quantified by flow cytometry using CYTO-ID Autophagy detection kit. As autophagy is a dynamic process, we added chloroquine (CQ) to block autophagosome fusion with lysosomes, inhibiting the degradation of the autophagosomes and their cargo. In CQ-free conditions, after 48 h of the decidualization treatment, we found no differences in CYTO-ID intensity between decidualized (D) and non-decidualized (ND) cells (Figure 1). On the contrary, in CQ-blocked conditions, the CYTO-ID intensity significantly increased in D cells in comparison to D-CQ-free, ND-CQ-free, and ND-CQ-blocked conditions (Figure 1). 

These results indicate that autophagosomes are present in t-HESC in the non-decidualized state with no detectable AF (Figure 1, ND-CQ-free versus ND-CQ-blocked), and that autophagy is activated and AF is increased during decidualization (Figure 1, D-CQ-free versus D-CQ-blocked).

### 2.2. The Knockdown of ATG5 Is More Effective Than the Knockdown of ATG7 to Impair Autophagy During Decidualization

Next, we wanted to define whether the observed increase in AF is required for a proper decidualization. One experimental approach to evaluate the importance of autophagy is to use small interference RNA (siRNA) to knockdown specific genes of the autophagy core machinery. To this end, we transfected cells with siRNA against two ATG proteins, ATG7 and ATG5. Both of these proteins are involved in the ubiquitin-like conjugation systems needed for autophagosome formation and are widely chosen as targets for the study of autophagy in knockdown and knockout experimental models [14,21,22,23,24].

**Figure 2:**
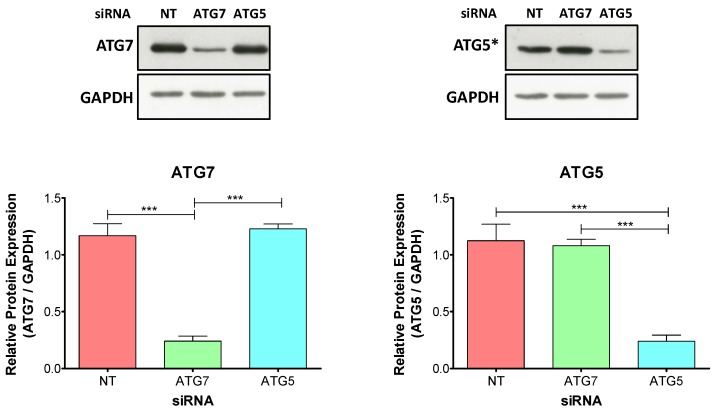
Transfection of ATG7 and ATG5 specific siRNA into t-HESC: down-regulation of specific targets. t-HESC were transfected with siRNA against ATG7, ATG5 or non-target for 24 h. After transfection, cells were treated with 2%-FBS medium with MPA + E + 8-Br-cAMP for further 48 h. The figure shows representative immunoblots for protein levels of ATG7, ATG5, and GAPDH (internal control) (*n* = 4). Data represent mean relative protein expression to GAPDH ± SEM. *** *p* < 0.001 by one-way ANOVA followed by Tukey test. t-HESC: immortalized human endometrial stromal cells; MPA: medroxyprogesterone; E: estradiol; 8-Br-cAMP: 8-Bromo-cyclic adenosine monophosphate; siRNA: small interfering RNA; NT: non-target. *The band corresponding to ATG5* represents the detection of ATG5 conjugated to ATG12 (ATG5-ATG12).

First, we evaluated whether the transfections with specific siRNA were effective to downregulate the corresponding proteins. Immunoblot analysis showed a specific downregulation of ATG7 and ATG5 by the corresponding siRNA after 48 h of the decidualization treatment compared to non-target-siRNA (NT-siRNA) transfected cells (Figure 2).

**Figure 3:**
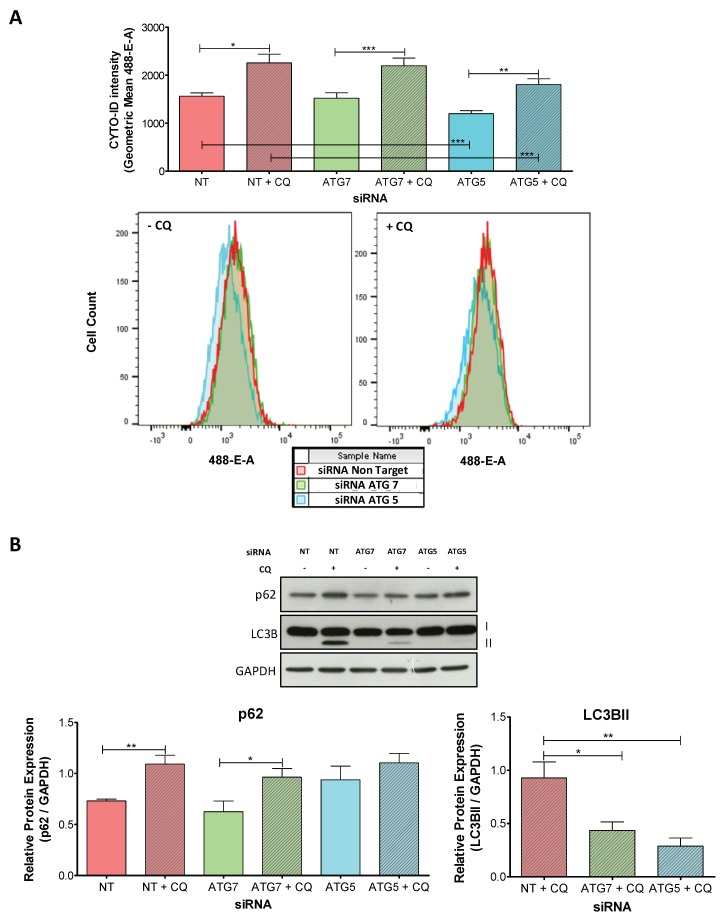
Transfection of ATG7 and ATG5 specific siRNA into t-HESC: impairment of autophagic flux. t-HESC were transfected with siRNA against ATG7, ATG5 or non-target for 24 h. After transfection, cells were treated with 2%-FBS medium with MPA + E + 8-Br-cAMP for further 48 h. Chloroquine (25 µM) was added 6 h before harvesting the cells. (**A**) Flow Cytometry analysis of t-HESC cells stained with CYTO-ID Autophagy detection kit. Data represent the geometric mean of CYTO-ID intensity ± SEM (*n* = 4). Histograms show representative cell count profiles. (**B**) The figure shows representative immunoblots for protein levels of p62, LC3B, and GAPDH (internal control) (*n* = 5). Data represent mean relative protein expression to GAPDH ± SEM. * *p* < 0.05; ** *p* < 0.01; *** *p* < 0.001 by Paired Student’s *t*-test or one-way ANOVA followed by Tukey test. CQ: chloroquine; t-HESC: immortalized human endometrial stromal cells; MPA: medroxyprogesterone; E: estradiol; 8-Br-cAMP: 8-Bromo-cyclic adenosine monophosphate; siRNA: small interfering RNA; NT: non-target.

We then assessed whether the knockdowns of ATG7 and ATG5 were effective to impair autophagy during decidualization. We measured the changes in AF in siRNA-transfected D cells using CYTO-ID Autophagy detection kit. Our results showed that AF is active in all D cells independently of the transfected siRNA (Figure 3A, CQ-free versus CQ-blocked conditions for each siRNA). However, the overall intensity is decreased in D cells transfected with ATG5-siRNA compared to those transfected with NT-siRNA (NT-siRNA-D-CQ-free versus ATG5-siRNA-D-CQ-free cells and NT-siRNA-D-CQ-blocked versus ATG5-siRNA-D-CQ-blocked cells) indicating a reduced AF (Figure 3A). In contrast, ATG7 knockdown had no significant effect on AF compared to NT-siRNA-transfected D cells (Figure 3A).

To complement the observed effect on AF, we evaluated the protein levels of p62, an autophagy receptor, and of LC3B after 48 h of the decidualization treatment (Figure 3B). LC3B belongs to the ubiquitin-like mammalian ATG8 family of proteins (LC3 and GABARAP proteins) and is synthesized as a pro-LC3B precursor. ATG4 processes pro-LC3B protein into LC3BI, that is later lipidated in a ubiquitin-like reaction by specialized enzymes that include ATG7. LC3BII is the lipidated version of LC3BI and is always associated with the autophagosome membrane [25]. As p62 and LC3BII are degraded during autophagy, the comparison between CQ-free and CQ-blocked conditions is used to quantify differences in AF [21,26].

Our results showed that in NT and ATG7-siRNA-transfected D cells, p62 is degraded at similar levels, confirming that there are no detectable differences in AF between these two conditions. In contrast, ATG5 knockdown inhibited p62 degradation, as seen in the elevated protein detection, further indicating an impaired AF in ATG5-siRNA-transfected D cells (Figure 3B). Regarding LC3B, the band corresponding to LC3BII was not present in CQ-free conditions for all the different siRNA but was present in CQ-blocked conditions indicating active AF. However, when we compared the relative expression of LC3BII to GAPDH, we detected a decrease in LC3BII levels with both ATG proteins specific siRNA compared to NT-siRNA-transfected D cells, that was particularly prominent in ATG5-siRNA-transfected D cells (Figure 3B).

Taken together, these results indicate that ATG5-siRNA is more effective than ATG7-siRNA to impair autophagy during t-HESC decidualization. While ATG5-siRNA-transfected D cells had a decrease in AF and a reduction in p62 degradation and in LC3BI lipidation, ATG7-siRNA-transfected cells only had a reduced lipidation of LC3BI.

### 2.3. Knockdowns of ATG7 and ATG5 Impair Decidualization

**Figure 4:**
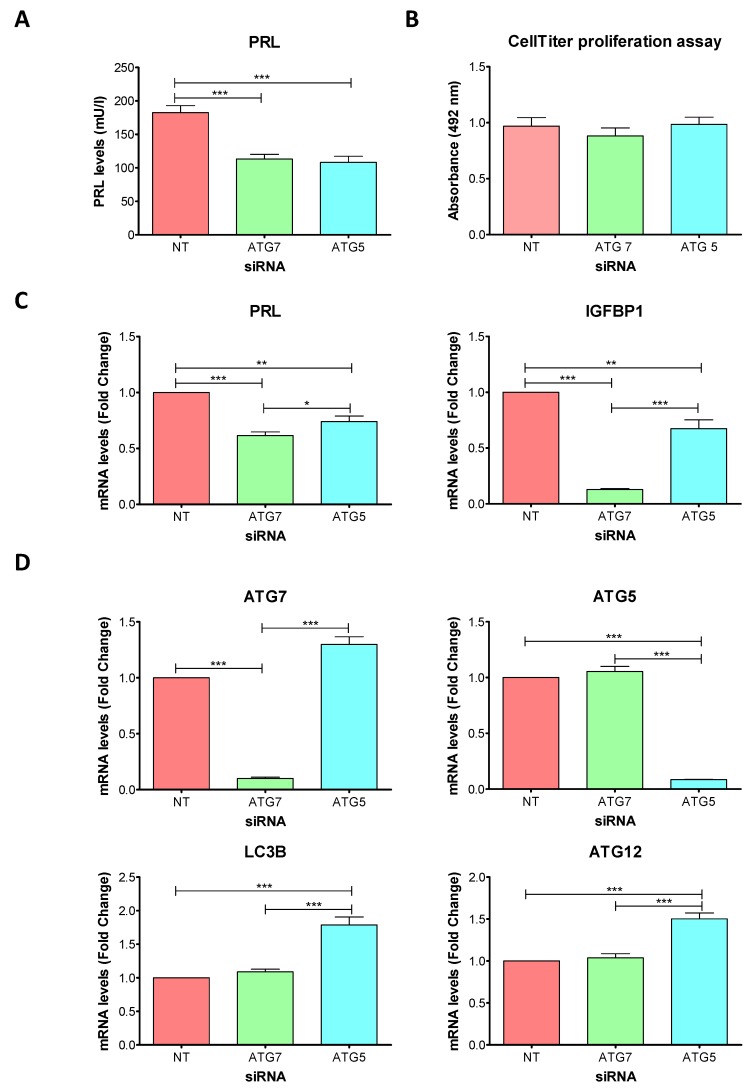
Transfection of ATG7 and ATG5 specific siRNA into t-HESC: effect on decidualization. t-HESC cells were transfected with siRNA against ATG7, ATG5 or non-target for 24 h. After transfection, cells were treated with 2%-FBS medium with MPA + E + 8-Br-cAMP for further 96 h. (**A**) Prolactin (PRL) levels in the cell culture supernatant (*n* = 8). (**B**) Cell proliferation was evaluated by CellTiter proliferation assay. Data represent mean Absorbance (492 nm) ± SEM (*n* = 4). (**C**) PRL and Insulin-like growth factor binding protein 1 (IGFBP1) mRNA expression levels analyzed by RT-PCR. Data represent mean fold change ± SEM (*n* = 5). (**D**) ATG7, ATG5, ATG12, and LC3B mRNA expression levels analyzed by RT-PCR. Data represent mean fold change ± SEM (*n* = 5). * *p* < 0.05; ** *p* < 0.01; *** *p* < 0.001 by one-way ANOVA followed by Tukey test. t-HESC: immortalized human endometrial stromal cells; MPA: medroxyprogesterone; E: estradiol; 8-Br-cAMP: 8-Bromo-cyclic adenosine monophosphate; siRNA: small interfering RNA; NT: non-target.

To evaluate the impact of ATG7 and ATG5 knockdowns during decidualization, we measured the protein expression level of PRL in the cell culture supernatant (SN) of siRNA-transfected cells after 96 h of decidualization treatment. PRL protein levels decreased to similar levels in both ATG-siRNA-transfected D cells compared to NT-siRNA-transfected D cells (Figure 4A).

To define if the observed decrease in PRL levels were due to differences in cell number, we evaluated whether cell proliferation was affected by ATG-siRNA transfection. The CellTiter proliferation assay verified that there were no significant differences in cell number between siRNA-transfected D cells. (Figure 4B).

Then, we quantified the mRNA levels of PRL and IGFBP1. The mRNA levels of PRL in siRNA-transfected D cells were similar to those observed for PRL protein in the cell culture supernatant, showing that both ATG-siRNAs were effective to decrease PRL levels after 96 h of the decidualization treatment (Figure 4C). However, compared to ATG5-siRNA, ATG7-siRNA led to a stronger decrease in PRL mRNA expression. The mRNA levels of IGFBP1 were also decreased in ATG-siRNA-transfected D cells, and again, the decrease observed after ATG7-siRNA transfection was stronger than the decrease observed by ATG5-siRNA transfection (Figure 4C).

Finally, we evaluated the mRNA levels of ATG5, ATG7, ATG12 and LC3B (Figure 4D). After 96 h of the siRNA transfection, the expression of ATG7 and ATG5 was still decreased and this decrease was specific according to the transfected siRNA. The expression levels of ATG12 and LC3 were not modified by ATG7-siRNA. In contrast, in ATG5-siRNA-transfected D cells the mRNA levels of ATG12 and LC3B, as well as ATG7 mRNA levels, were increased compared to NT and ATG7-siRNA-transfected D cells.

These results indicate that the knockdowns of ATG7 and ATG5 led to lower expression levels of PRL and IGFBP1, indicating an impaired decidualization of t-HESC.

## 3. Discussion

Our results showed that autophagy is activated during the decidualization of t-HESC and that the impairment of autophagy by ATG7 and, in particular, by ATG5 knockdowns led to lower levels of expression of PRL and IGFBP1, indicating a defective decidualization.

### 3.1. Possible Roles of Autophagy during Decidualization

As demonstrated for the differentiation and regulation of cellular fate in several cell types during mammalian early development and adult life, active autophagy could contribute to a proper decidualization in different ways [8,27]. For example, through autophagy, the intracellular composition can be easily modified, allowing the old ESC identity to be recycled into the new decidual identity. In addition, autophagy could contribute to a fast clearance of mRNA and nascent proteins synthesized in response to the program of gene expression of ESC, contributing to the complex change in gene expression that takes place during decidualization. This clearance function of autophagy could also apply for the recycling of damaged organelles. For example, proper elimination of damaged mitochondria could contribute to a reduction of reactive oxygen species (ROS) and also regulate the release of proapoptotic factors [28,29].

From the point of view of energetic needs, autophagy could be important to deal with the high energetic requirements that the decidual program demands by helping to recycle metabolites that would need energy-expensive biosynthesis. Furthermore, autophagy could contribute to the survival of decidual cells under the low oxygen and low-nutrient conditions of the feto-maternal interface, as it has been suggested for the invasion of extravillous trophoblasts [30].

Impaired autophagy, affecting all the processes stated above, could lead to a defective decidualization, compromising pregnancy. Furthermore, impaired pregnancy establishment and progression has been associated with age-related defective decidualization [31], and in a parallel pathological reduction in the autophagy potential has also been related to aging [12]. This could suggest a possible connection between impaired decidualization and aging trough defective autophagy.

### 3.2. Knockdowns of ATG7 and ATG5: Autophagy-Dependent and Autophagy-Independent Roles During Decidualization?

Our experiments with ATG7 and ATG5 knockdowns led to a defective decidualization of t-HESC. However, there was a difference regarding the level of impairment of autophagy for each ATG-siRNA transfected.

ATG5 was more effective than ATG7 to impair autophagy during decidualization, as cells transfected with ATG5-siRNA showed impaired autophagy by the three different methods we used to measure AF (CYTO-ID Autophagy detection kit and p62 and LC3BII protein detection levels). These results suggest that the defect decidualization resulted due to ATG5 knockdown could be directly related to a “quantity issue”: lower levels of autophagy in ATG5-siRNA-transfected D cells. Furthermore, the upregulation of ATG7, LC3B, and ATG12 mRNA levels observed in ATG5-siRNA-transfected D cells could represent a compensatory response.

Regarding ATG7, we observed impaired autophagy during decidualization only when we analyzed the levels of LC3BII. However, we observed a strong downregulation of decidualization markers indicating that ATG7 knockdown affects decidualization even more than the knockdown of ATG5. We have three hypotheses to explain the observed results. First, ATG7 function during autophagy is tightly related to the ubiquitin-like lipidation of the members of the LC3 and GABARAP family of proteins. It has been described that LC3 and GABARAP family members interact with different autophagy receptors for successful cargo sequestration [32,33]. In ESC, we believe that ATG7 downregulation could be affecting the selective nature of autophagy rather the AF. Therefore, a “quality issue” that leads to the wrong clearance of targeted cargos could dysregulate cellular homeostasis in ESC, affecting decidualization. Second, there is increasing evidence that ATG proteins have autophagy-independent roles [34,35,36]. We think that ATG7 knockdown could affect decidualization by a, so far, unknown function. Finally, In addition to the canonical autophagy pathways, there are evidences regarding several non-canonical autophagy pathways [37,38]. We, therefore, suggest that the knockdown of ATG7 could lead to the activation of compensatory non-canonical autophagy pathways during decidualization, explaining why AF was not affected while decidualization was more affected than with ATG5 knockdown. Results seen in ATG5 knockdown could similarly be related to these hypotheses.

### 3.3. Autophagy and Endometrial Physiology

Autophagy is a complex cellular process. In addition to the different types of autophagy, a lot of new functions and interactions for ATG proteins are being discovered, indicating how fast the autophagy field is growing. We believe that the understanding of autophagy within endometrial physiology, in particular regarding decidualization, will also grow in the future years. Researchers should be cautious when organizing their experiments, as autophagy is a dynamic process, and static measurements could lead to misinterpretation of the results [21]. We are aware that further experiments are needed to define the exact role of ATG7, ATG5, and autophagy during endometrial decidualization. However, we are positive that the findings of this work will lead to defining autophagy as a new field of study within the scientific research regarding endometrial physiology, leading to a better understanding of the endometrial function and facilitating the identification and selection of new therapeutic targets for endometrial pathologies.

## 4. Materials and Methods

### 4.1. Cell Culture and in Vitro Decidualization Experiments

#### 4.1.1. Cell Culture

Human immortalized endometrial stromal cells (t-HESC, ATCC CRL-4003) were purchased from LGC (LGC Standards GmbH, Wesel, Germany) and cultivated in DMEM-F12 medium (D2906, Sigma-Aldrich, Taufkirchen, Germany) supplemented with 10% of Charcoal/Dextran treated fetal bovine serum (FBS) (HyClone, GE Healthcare Europe, Freiburg, Germany) according to ATCC recommended instructions.

#### 4.1.2. Decidualization Experiments

t-HESC were trypsinized, counted, and plated in 6-well plates at a density of 100,000 cells/well. After 24 h medium was removed and cells were rinsed with PBS. Then, cells were treated with 2%-FBS DMEM-F12 medium supplemented with 1 µM Medroxyprogesterone (MPA, Sigma–Aldrich, St. Louis, MO, USA), 10 nM Estradiol (E, Sigma–Aldrich, St. Louis, MO, USA) and 0.5 mM 8-Bromo-cAMP (8-Br-cAMP, Sigma–Aldrich, St. Louis, MO, USA) (Decidualized cells, D) or vehicle solutions (non-decidualized cells, ND). Cells were harvested after 48 h for autophagic flux evaluation. For AF detection, chloroquine (25 µM) (C6628, Sigma-Aldrich, Taufkirchen, Germany) or vehicle solution were added to culture medium 6 h before harvesting the cells.

### 4.2. Knockdown Experiments: Small Interfering RNA (siRNA) Transfection Followed by in Vitro Decidualization Experiments

#### 4.2.1. siRNA Transfection

t-HESC were trypsinized, counted and plated in 6-well plates at a density of 75,000 cells/well (for autophagic flux evaluation and for total protein and RNA extraction) or in 96-well plates at a density of 500 cells/well (for proliferation assays). After 24 h, the medium was removed, and cells were rinsed with PBS. Then 1 mL of 5% FBS DMEM-F12 was added to each well of the 6-well plates or 100 µL of 5% FBS DMEM-F12 to each well of the 96-well plates. Finally, 500 µL (6-well plates) or 50 µL (96-well plates) of Opti-MEM (11058-021, Gibco, Karlsruhe, Germany) containing siRNA against ATG7 or ATG5 or non-target proteins and 5 µL (6-well plates) or 0.5 µL (96-well plates) of Lipofectamin RNAiMAX Transfection Reagent (Invitrogen, Life Technologies GmbH, Darmstadt, Germany) were added to each well. All siRNA (listed in Supplemental Table S1) were purchased from Dharmacon (Horizon Discovery Ltd., Cambridge, UK) and the final concentration used was 30nM. siRNA transfection was performed for 20 to 24 h.

#### 4.2.2. Decidualization Experiments Following siRNA Transfection

After transfection, cells were treated with 2%-FBS DMEM-F12 medium supplemented with 1 µM MPA, 10 nM E, and 0.5 mM 8-Br-cAMP (D cells) or vehicle solutions (ND cells). The medium was changed every 48 h. Cell supernatants and cell samples were collected after 48 or 96 h of the decidualization treatment. siRNA knockdown of specific targets was corroborated at the protein and mRNA levels by Western blot and real time-PCR (RT-PCR), respectively. For AF detection, chloroquine (25 µM) or vehicle solution were added to culture medium 6 h before harvesting the cells.

### 4.3. Flow Cytometry Autophagic Flux Measurement

Live cells were collected after 48 h of the decidualization treatment. For AF detection, chloroquine (25 µM) or vehicle solution were added to culture medium 6 h before harvesting the cells. Cells were stained with CYTO-ID Autophagy detection kit 2.0 (Enzo Life Sciences, Farmingdale, NY, USA) according to manufactures instructions. Briefly, cells were trypsinized, centrifuged at 1000 rpm for 5 min and washed in 5% FBS-PBS. Then, cells were collected by centrifugation and resuspended in 250 μL of 5% FBS-PBS. After that, 250 μL of CYTO-ID Green staining solution (1:1000 in 5% FBS-PBS) was added to the cells. Incubation was performed for 30 min at room temperature in the dark. Gently pipetting of the cells was performed during the incubation to achieve mono-disperse cell suspension. Cells were collected by centrifugation and washed with 5% FBS-PBS. Cells were resuspended in 500 μL of 5% FBS-PBS and analyzed by flow cytometry (BD FACSCanto II, Flow Cytometry & FACS Core Facility, ZMBH, Heidelberg). Unstained cells were used to set up background fluorescence.

### 4.4. Western Blot Analysis for Protein Detection

Cell extracts were collected in RIPA buffer (89900, Thermo Scientific, Life Technologies GmbH, Darmstadt, Germany) supplemented with EDTA-free Complete Protease Inhibitor (Roche, Mannheim, Germany) and PhosSTOP Phosphatase Inhibitor Cocktail (Roche, Mannheim, Germany) after 48 h of the decidualization treatment. Sodium dodecyl sulfate-polyacrylamide gel electrophoresis (SDS-PAGE) followed by immunoblot analysis was performed to detect ATG7 (1:1000, rabbit monoclonal antibody #8558, CST, Cell Signaling Technology Europe, Frankfurt, Germany), ATG5 (1:1000, rabbit polyclonal antibody #2630, CST), p62 (1:1000, rabbit polyclonal antibody #5114, CST), LC3B (1:1000, rabbit polyclonal antibody #2775, CST), and GAPDH (1:2000, mouse monoclonal antibody sc-47724, Santa Cruz Biotechnology Inc, Dallas, TX, USA). Mouse anti-rabbit IgG-HRP (1:8000, sc-2357, Santa Cruz Biotechnology) and donkey anti-mouse IgG-HRP (1:10000, sc-2318, Santa Cruz Biotechnology) were used as secondary antibodies. Band detection was performed with ECL Prime Western Blotting Detection Reagents (RPN2232, Amersham, GE Healthcare, Buckinghamshire, UK) and Carestream BIOMAX MR Film (Carestream Health Inc, NY, USA). Films were air dried and scanned. Band intensities were measured with ImageJ software (National Institute of Health, Bethesda, Maryland, USA). Relative band intensity to GAPDH signal was compared between treatments.

### 4.5. Prolactin Quantification

Cell culture supernatants were collected after 96 h of the decidualization protocol. Prolactin protein levels were quantified in the SN by double sandwich assay ELISA (Siemens Diagnostics, Eschborn, Germany) according to the manufacturer’s instruction.

### 4.6. Gene Expression Assays

Cell extracts were collected in TRIzol Reagent (Invitrogen, Life Technologies GmbH, Darmstadt, Germany) after 96 h of the decidualization treatment. Total RNA extraction was performed according to the TRIzol manufacturer’s instructions. RNA was quantified (NanoDrop, ND-1000) and 1 µg of total RNA was reverse transcribed with the Reverse Transcription System (Promega Corporation, Madison, WI, USA). RT-PCR was performed using Tagman primers (listed in Supplemental Table S1) (Life Technologies GmbH, Darmstadt, Germany) and TaqMan universal PCR master mix (Applied Biosystems, Life Technologies GmbH, Darmstadt, Germany). RT-PCR reactions were run in the Fast Forward 7500 real-time PCR-system (Applied Biosystems, Life Technologies GmbH, Darmstadt, Germany), and results were analyzed according to the ΔΔCt method [39]. RPLP0 was used as internal control for the normalization of the Ct values. Normalized Ct values (ΔCt) were used for statistical analysis.

### 4.7. Proliferation Assay

Proliferation assay was performed for siRNA-transfected cells after 96 h of the decidualization treatment. For CellTiter 96 Aqueous One Solution Cell Proliferation assay (Promega Corporation, Madison, WI, USA) 20 µL of CellTiter 96 Aqueous One Solution was added to each well of the 96-well plates and incubated at 37 °C for 2 h. After incubation, absorbance was measured at 492 nm.

### 4.8. Statistical Analysis

Paired Student’s *t*-test and one-way analysis of variance (ANOVA) followed by Tukey Multiple Comparison Test were used for statistical analysis. Differences between treatments were considered significant when *p* < 0.05. Statistical Analysis was carried out using GraphPad Prim 4.0 (GraphPad Software Inc., La Jolla, CA, USA).

## 5. Conclusions

In conclusion, in this work, we describe a previously unknown connection between autophagy and endometrial physiology. We show, for the first time, that autophagy is increased during decidualization of t-HESC and we further show that the impairment of autophagy leads to a defective decidualization. Moreover, we suggest that ATG7 and ATG5 may regulate autophagy in endometrial stromal cells at different levels. While ATG5 knockdown could be related to an autophagy quantity issue, directly affecting the rate of autophagic flux, ATG7 knockdown effect could be more related to a quality issue, affecting autophagy cargo, or to an ATG7 autophagy-independent role.

## Supplementary Materials

Supplementary materials can be found at https://www.mdpi.com/1422-0067/20/12/3066/s1.

## Abbreviations

8-Br-cAMP	8-Bromo-cyclic adenosine monophosphate
AF	Autophagic flux
ATG	Autophagy-related
CQ	Chloroquine
D	Decidualized
E	Estradiol
ESC	Endometrial stromal cells
IGFBP1	Insulin-like growth factor binding protein 1
MPA	Medroxyprogesterone
ND	Non decidualized
NT	Non-target
PRL	Prolactin
siRNA	Small interfering RNA
SN	Supernatant
t-HESC	Immortalized human endometrial stromal cells
