# Supplementary material for: Decreased Autophagy Impairs Decidualization of Human Endometrial Stromal Cells: A Role for ATG Proteins in Endometrial Physiology
Source: Int J Mol Sci. 2019 Jun 23;20(12):3066. doi: 10.3390/ijms20123066 (PMC6628477; doi:10.3390/ijms20123066)
Supplement: Supplementary file 1 [file ijms-20-03066-s001.pdf]

		Gene	Catalog Number
**siRNA**	**ON-TARGET plus**	ATG7	L020112-00-0005
		ATG5	L004374-00-0005
		Non- target	D-001810-10-05
**Taqman primers**	**Decidualization markers**	PRL	hs00168730_m1
		IGFBP1	hs00236877_m1
	**Autophagy-related genes**	ATG7	hs00197348_m1
		ATG5	hs00169468_m1
		ATG12	hs01047860_g1
		MAP1LC3B (LC3B)	hs00797944_s1
	**Housekeeping gene**	RPLP0	hs99999902_m1

Supplemental Table S1. Detailed list of siRNA and primers used.
